# Ninety‐three point seven percent pooled 10‐year survival rate following lateral unicompartmental knee arthroplasty for isolated lateral knee osteoarthritis—A systematic review and meta‐analysis

**DOI:** 10.1002/jeo2.70870

**Published:** 2026-08-03

**Authors:** Tsvetan Tsenkov, Yasen Nikolov, Atanas Panev, Nikolay Dimitrov, Michael T. Hirschmann

**Affiliations:** ^1^ Department of Orthopaedics and Traumatology Medical University – Sofia Sofia Bulgaria; ^2^ University Hospital of Orthopaedics “Prof. B. Boichev” Sofia Bulgaria; ^3^ Department of Orthopaedic Surgery and Traumatology Kantonsspital Baselland (Bruderholz, Liestal, Laufen) Bruderholz Switzerland; ^4^ University of Basel Basel Switzerland

**Keywords:** knee arthroplasty, lateral knee OA, lateral UKA, osteoarthritis, unicompartmental, unicondylar

## Abstract

**Purpose:**

To systematically review the available evidence on long‐term outcomes following lateral unicompartmental knee arthroplasty in lateral knee osteoarthritis.

**Methods:**

An advanced search was done throughout three electronic databases (PubMed, Scopus and Embase) involving the words ‘lateral unicompartmental knee arthroplasty’ and ‘lateral knee osteoarthritis’. Inclusion and exclusion criteria were set. The Newcastle‐Ottawa scale was used in assessing the study quality. Extracted were study demographics, survival rate, follow‐up period, indications and contraindications, implant type, revision rate and outcome tools. The data were systematically collected and meta‐analysed.

**Results:**

After an initial screening of 1864 articles, 15 articles were selected for the review. The study quality was evaluated as ‘high’ in most of the selected articles. The pooled 10‐year survival rate was 93.7% (95% CI: 92.5%−94.9%). The mean follow‐up period ranged between 7 and 14 years (pooled mean follow‐up 9.1 years). A significant negative correlation was found between patient BMI and survival rate (*p* = 0.03). Female participants were prevalent among the studies; however, no strong evidence linked the male proportion and survival rate (*p* > 0.05). The pooled revision rate was 4.6%.

**Conclusions:**

Lateral unicompartmental knee arthroplasty for isolated lateral knee osteoarthritis provided a high long‐term survival rate with a low 10‐year revision rate. This was provided in a non‐ACL‐deficient knee with a preoperative flexion of above 90 degrees and a correctable valgus by using a fixed‐bearing implant. Females were more likely to undergo this procedure. A negative correlation was found between patient BMI and survival rate. There was a notable variability in the reported outcome measures among studies.

**Level of Evidence:**

Level IV, systematic review of therapeutic studies.

**Abbreviations**
BMI: body‐mass index
CI: confidence interval
HA: Hemophilia
HC: Hemochromatosis
IA: inflammatory arthritis
IKDC: International Knee Documentation Committee radiological criteria
LCCK: legacy constrained condylar knee
LoE: level of evidence
LPA: lateral parapatellar approach
LUKA: lateral unicompartmental knee arthroplasty
MD: mean difference
NOS: Newcastle Ottawa scale
OA: osteoarthritis
OR: odds ratio
ROM: range of movement
RR: revision rate
SD: standard deviation
SR: survival rate
TKA: total knee arthroplasty
UKA: unicompartmental knee arthroplasty

## INTRODUCTION

Isolated lateral compartment knee osteoarthritis (OA) is very rare, with an incidence of less than 10% [15, 46]. Knee arthroplasty is considered in such cases. This may be in the form of total knee arthroplasty (TKA) or a lateral unicompartmental knee arthroplasty (LUKA) [9]. LUKA offers the advantage of preserving better functional outcomes for the patient, as it involves minimal alteration to key anatomical structures, including the medial and lateral collateral ligaments, as well as the anterior and posterior cruciate ligaments, in comparison to TKA or the use of a legacy constrained condylar knee (LCCK) prosthesis [46].

Despite initial poor outcomes that have been reported in the literature, with advancements in implant design, surgical techniques and patient selection LUKA has gained a renewed interest among knee surgeons [24]. Recent studies have reported good clinical outcomes, decreased complications and morbidity and a lower necessity for morphine based pain medication at 4 weeks when compared to TKA [8, 11]. Biomechanical studies have also confirmed that the design of the unicompartmental knee arthroplasty (UKA) implants more closely mirrors normal knee kinematics when compared to TKA [2]. A fact which comes along with improved patient satisfaction, likely due to the perceived normal knee function. On the other hand, registry data have shown a higher rate of revision after primary UKA when compared to TKA [23, 27].

Multiple studies documented the success of LUKA, it is performed 10 times less than the medial UKA. LUKA reportedly accounts for 1% of all arthroplasty procedures. A recent meta‐analysis between medial and lateral UKA discovered that there was no statistical difference in survival, functional outcomes and pain relief between the two [18]. Several large case‐cohort studies exist in the literature, however, there are few long‐term follow‐up comparative studies and no systematic review on patients with isolated lateral knee OA, who have undergone LUKA with a long‐term follow‐up [10]. Bonanzinga et al. [7] performed a systematic literature review on mid‐term clinical outcomes and survival rates (SRs) from LUKA. The studies' mean follow‐up period was 5 years. Several long‐term case series have emerged in the literature since then. This study aims to investigate the long‐term outcomes of LUKA in patients with lateral knee OA.

## METHODS

The preferred reporting items for systematic reviews and meta‐analyses (PRISMA) checklist was followed when conducting this study. The Institutional Review Board (IRB) granted an exemption for this research. This study was registered in the PROSPERO register. The study review protocol is available in the native language of the main author and can be assessed upon reasonable request.

### Screening and study selection process

**Figure 1:**
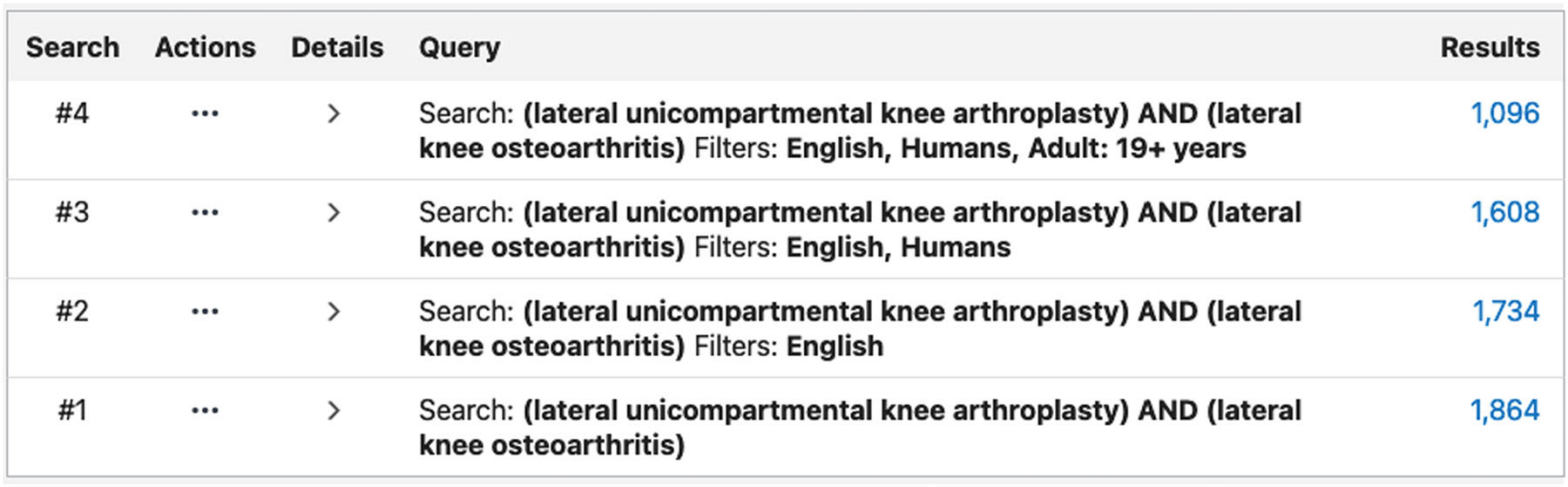
Search strategy for the study—Initial screening.

Three electronic databases were used (PubMed, Scopus and Embase), last updated in November 2024. The search strategy included an advanced search involving the words ‘lateral unicompartmental knee arthroplasty’ and ‘lateral knee osteoarthritis’. Custom filters were applied to the advanced search engine. The ‘Results by year’ option was set by default to include all the available studies up until 2024. The article language was set to English only. The ‘Human’ species option was checked, along with the age option of ‘Adults: 19+ years’. The projected number of studies (*n*) and the search parameters from the primary screening are visualised in Figure 1.

A custom display option was chosen where the whole study abstract was seen directly in scrolling mode. The keywords, the Level of Evidence (LoE), the journal name and the publication date were also visible from this option.

An extensive check of the remaining articles was then conducted by two authors independently. Duplicate papers, studies dealing with TKA, bi‐compartmental knee arthroplasty, medial UKA, studies about cementless knee arthroplasty and case reports were excluded. Furthermore, technical articles, narrative reviews and complication studies were excluded.

The remaining articles were screened for inclusion, and any discrepancies in the selection process were resolved through discussions among the reviewers. All articles reporting outcomes in patients with lateral knee OA treated with LUKA and a mean follow‐up period exceeding 6 years were included, as follow‐up durations up to 5 years are categorised as mid‐term follow‐up [9]. Only studies utilising standardised outcome measurement instruments were considered. These were then reviewed in full‐text by the two independent raters. The flow process diagram was created using the PRISMA 2020 flow diagram sample [38].

Data on the study and patient demographics, and outcomes were then extracted. This included the main author's name, year of publication, single‐ or multicenter involvement, number of patients and procedures included, the participant sex ratio, age, body mass index (BMI), implant type, indications and contraindications for surgery, surgical approach, mean follow‐up, revision rate, preoperative and postoperative outcome tools and Kaplan–Meier survival estimates.

The Newcastle Ottawa scale (NOS) was used for the assessment of study quality. NOS is a convenient tool developed for quantitatively evaluating nonrandomized studies to be used in a systematic review [44]. The NOS has a maximum of nine points (best possible study quality). Papers with a NOS score of below five points were scored ‘low quality’, between six and seven points—medium quality and papers of eight and nine points were scored ‘high’ quality.

### Statistical methods

Review Manager 5.4 software and Microsoft Office Excel Version 16.48 were used in constructing the systematic review and meta‐analysis. The odds ratio (OR) and 95% confidence intervals (CI) were calculated for binary variables. The mean difference (MD) and 95% CI were used for continuous variables. Inverse variance weighting was used to calculate pooled estimates. Heterogeneity was assessed by using chi‐square‐based *Q* and *I* ^2^ values. *P* > 0.10 or *I* ^2^ < 50% indicated no study heterogeneity, then we used a fixed‐effects model. A random‐effects model was used with significant heterogeneity studies. *P* < 0.05 was considered as a statistically significant difference. Pearson's correlation coefficient was used to assess whether there was a correlation between the patient's age at the point of surgery and the SR.

## RESULTS

**Figure 2:**
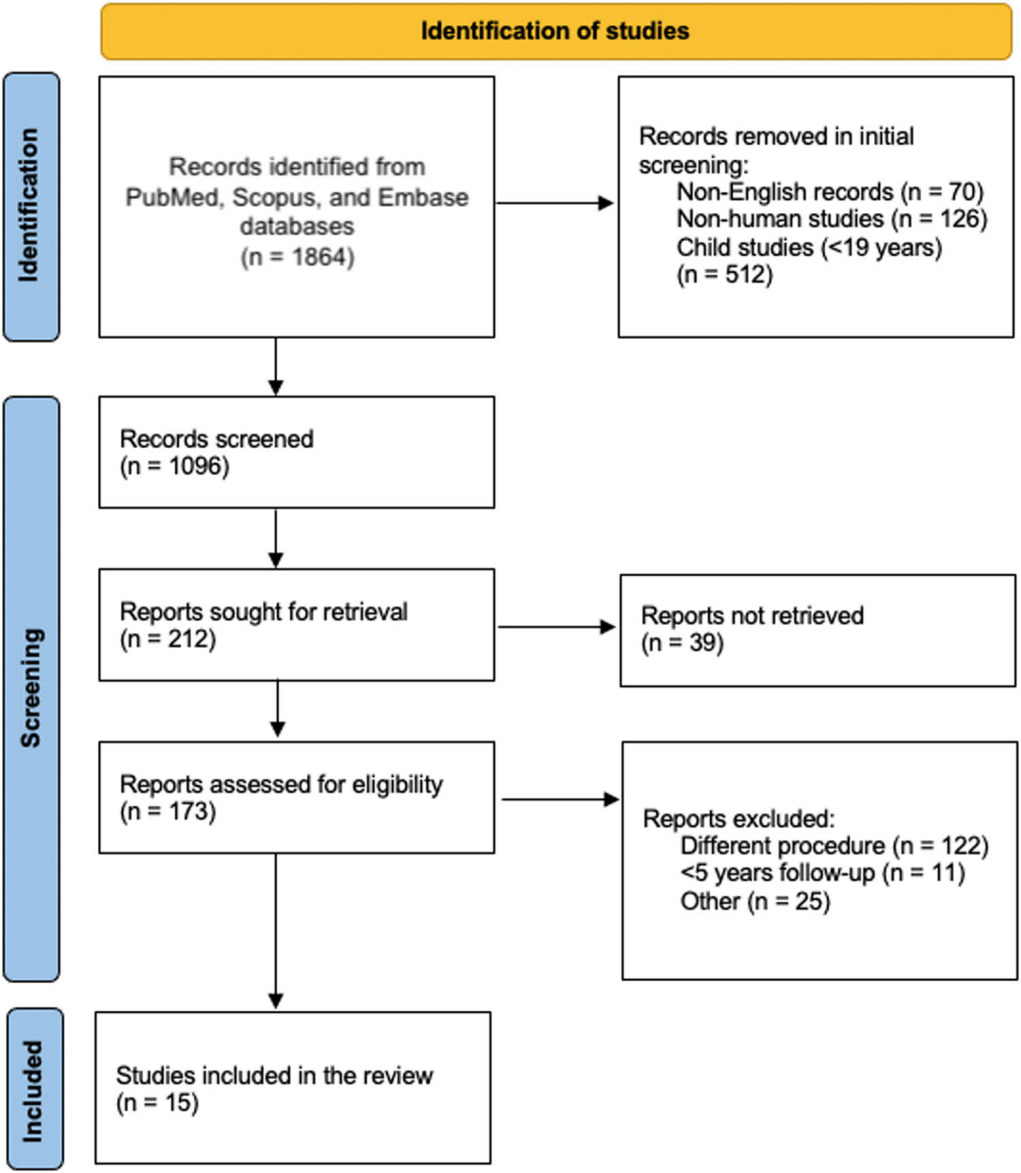
Flow diagram.

A total of 1864 relevant articles were retrieved following the search strategy. A total of 768 articles were removed during the additional filtering process, and 1096 records were screened again. When implementing the inclusion and exclusion criteria, a total of 173 reports were assessed for eligibility. Following a thorough review of the full‐text articles and an additional examination of the references within the retrieved articles, a total of 15 articles were ultimately included in the review [3, 5, 12, 13, 14, 20, 21, 25, 30, 33, 35, 37, 39, 40, 42]. The literature screening results and study selection are shown in Figure 2.

This review included three level III studies and 12 level IV studies. The quality of the studies was assessed as ‘high’ in 93% (*n* = 14) of the articles based on the Newcastle‐Ottawa Quality Assessment Scale.

The mean follow‐up period ranged from 7 to 14 years with a pooled mean of 9.1 years (95%CI: 7.6–10.5) (Table 1).

**Table 1:** Study follow‐up period and inverse‐variance weighting.

Study	Follow‐up	Min	Max	*N*	SD	Variance	Weight	Weighted mean
Deroche et al. [12]	9.1	5	23	268	4.5	20.25	0.05	0.45
Harkin et al. [20]	10	1	22	161	5.3	27.56	0.04	0.36
Kennedy et al. [25]	7	3	14	325	2.8	7.56	0.13	0.93
Plancher et al. [40]	10	4	17	63	3.3	10.56	0.09	0.95
Montilla et al. [35]	8.2	2	12	42	2.5	6.25	0.16	1.31
Hartman et al. [21]	8.8	2	12	49	2.5	6.25	0.16	1.41
Mekkawy et al. [33]	7.6	5	14	53	2.3	5.06	0.20	1.50
Ashraf et al. [5]	9	2	21	88	4.8	22.56	0.04	0.40
Edminston et al. [13]	6.8	4	10	65	1.5	2.25	0.44	3.02
Lustig et al. [30]	14.2	10	18	46	2.0	4.00	0.25	3.55
Argenson et al. [3]	12.6	3	23	40	5.0	25.00	0.04	0.50
Ohdera et al. [37]	8.2	5	16	38	2.8	7.56	0.13	1.08
Pennington et al. [39]	12.4	3	16	29	3.3	10.56	0.09	1.17
Fitzsimons et al. [13]	6.6	2	11	255	2.3	5.06	0.19	1.30

Abbreviations: *N*, number of procedures; SD, standard deviation.

The majority of the studies were conducted at a single institution, as determined through cross‐referencing the names of the institutions and authors, comparing sample sizes and study dates, and noting the absence of explicitly stated overlaps. The number of patients (procedures) ranged from 29 (29) to 308 (325). The mean age ranged from 54 to 72 years, and the mean BMI in the studies was reported from 24.8 to 28 kg/m^2^. The females were significantly more than the males (*Z*‐score 6.87; *p* < 0.005). The male‐to‐female ratio ranged from 0.14 to 0.96. The mean reported SR ranged from 6.8 to 14.2 years. The most reported surgical approach was the lateral parapatellar approach (LPA). Two studies reported using a medial approach [3, 13], and one study reported a ‘minimal lateralization approach’ [35]. The basic characteristics of the included articles are shown in Table 2.

**Table 2:** Study characteristics.

Author	Year	Study	LoE	NOS score	*N*	M/F ratio	Implant type	SR (years)	RR (*n*)	Follow‐up (range)
Deroche et al. [12]	2020	Multi‐centre	IV	9	252 (268)	0.31	Fixed‐bearing	85.4% (10)	14.5% (39)	9.1 (5– 23)
								79.4% (20)		
Harkin et al. [20]	2024	Single‐centre	IV	9	153 (161)	0.73	Fixed‐bearing	98% (5)	6.8% (11)	10 (1–22)
								96% (10)		
								94.5% (15)		
Kennedy et al. [25]	2020	Single‐centre	IV	9	308 (325)	0.48	Mobile‐bearing	92.1% (5)	9.6% (34)	7 (3–14)
								84.6% (10)		
Plancher et al. [40]	2022	Single‐centre	III	8	61 (63)	0.47	Fixed‐bearing	99% (5)	4.9% (3)	10 (4–17)
								98% (10)		
Montilla et al. [35]	2024	Single‐centre	IV	9	40 (42)	0.4	Fixed‐bearing	100% (5)	0%	8.2 (2–12)
Hartman et al. [21]	2023	Single‐centre	III	7	49 (49)	0.81	Both	86% (10)	8.2% (4)	8.8 (2–12)
Mekkawy et al. [33]	2023	Single‐centre	IV	8	50 (53)	0.56	Fixed‐bearing	98% (5)	0.5% (1)	7.6 (5– 14)
Sangaletti et al. [42]	2024	Single‐centre	IV	9	40 (40)	0.6	Fixed‐bearing	93.1% (10)	7.5% (3)	11.1
Ashraf et al. [5]	2002	Single‐centre	IV	9	79 (88)	0.16	Fixed‐bearing	83% (10)	17% (15)	9 (2–21)
								74.5% (15)		
Edmiston et al. [13]	2018	Single‐centre	III	9	65 (65)	0.5	Fixed‐bearing	94% (7)	6.2% (4)	6.8 (4–10)
Lustig et al. [30]	2014	Single‐centre	IV	9	46 (46)	0.2	Fixed‐bearing	91.4% (15)	15.2% (7)	14.2 (10–18)
Argenson et al. [3]	2008	Single‐centre	IV	9	38 (40)	0.63	Fixed‐bearing	92% (10)	12.5% (5)	12.6 (3–23)
								84% (16)		
Ohdera et al. [37]	2001	Single‐centre	IV	8	37 (38)	0.27	Both	N/S	2.6% (1)	8.2 (5–16)
Pennington et al. [39]	2006	Single‐centre	IV	8	29 (29)	0.14	Fixed‐bearing	N/S	0%	12.4 (3–16)
Fitzsimons et al. [13]	2023	Single‐centre	IV	9	255 (255)	0.96	Fixed‐bearing	98.4% (5)	1.6% (4)	6.6 (2–11)

Abbreviations: LoE, level of evidence; M/F, male‐to‐female; N, number of patients (number of procedures); NOS, Newcastle‐Ottawa Scale; RR, revision rate; SR, survival rate.

### Indications and contraindications

The primary indication was isolated lateral knee OA; however, the authors also detailed specific indications and contraindications. However, the authors have reported specific indications and contraindications. They are presented in Table 3. Most of the authors report an active preoperative range of motion (ROM) of above 90 degrees as a main indication for LUKA, along with a reducible valgus deformity and a flexion contracture of less than 10 degrees. The main contraindications reported were symptomatic patellofemoral (PF) OA, anterior cruciate ligament (ACL) insufficiency and a fixed valgus deformity.

**Table 3:** Indications and contraindications for LUKA.

Author	Indications	Contraindications
Deroche et al. [12]	Ahlbäck grade ≥ 2	Symptomatic PF OA
	Flexion contracture < 10°	ACL insufficiency
		Medial OA
		IA
Harkin et al. [20]	Kellgren–Lawrence ≤ 2	Symptomatic PF OA
	RoM ≥ 90°	Simptomatic knee instability
	Flexion contracture < 15°	Medial OA
		IA, HC, HA
Kennedy et al. [25]	Correctable valgus	ACL insufficiency
	Lateral ‘bone‐on‐bone’ OA	Medial OA
Plancher et al. [40]	RoM ≥ 95°	Symptomatic PF OA
	Flexion contracture < 5°	Medial OA
		Fixed valgus > 15°
		Fixed flexion contracture > 15°
		Previous osteotomy, BMI > 40
Montilla et al. [35]	RoM ≥ 95°	Symptomatic PF OA
	Flexion contracture < 20°	ACL insufficiency
Sangaletti et al. [42]	RoM ≥ 90°	Symptomatic PF OA
	Flexion contracture < 10°	ACL insufficiency
	Lateral Kellgren–Lawrence ≥ 3	Fixed valgus > 15°
		Kellgren–Lawrence > 2 for medial
Ashraf et al. [5]	RoM ≥ 90°	Symptomatic PF OA
	Flexion contracture < 10°	Medial OA
Lustig et al. [30]	Lateral IKDC C or D	Symptomatic PF OA
	PF OA in patients > 70 years	ACL insufficiency
	Flexion contracture < 10°	Medial OA
		Patient > 85 kg
Argenson et al. [3]	Ahlbäck grade ≥ 2	Medial OA
	RoM ≥ 100°	
Ohdera et al. [37]	Lateral symptomatic OA	Rheumatoid arthritis
Pennington et al. [39]	RoM ≥ 90°	Kellgren–Lawrence > 2 for PF
	Flexion contracture < 10°	Kellgren–Lawrence > 2 for medial
	Chondrocalcinosis	ACL insufficiency
Fitzsimons et al. [13]	Correctable valgus	Symptomatic PF OA
		Medial OA
	Stable knee	Fixed flexion contracture > 5°
		IA

Abbreviations: ACL, anterior cruciate ligament; BMI, body mass index; HA, Hemophilia; HC, Hemochromatosis; IA, inflammatory arthritis; IKDC, International Knee Documentation Committee radiological criteria; LUKA, lateral unicompartmental knee arthroplasty; OA, osteoarthritis; PF, patellofemoral; RoM, range of motion.

### Implant type

Most of the studies reported results for a fixed‐bearing prosthesis (12 of 15 studies). One study reported the use of a mobile‐bearing type [25], and two studies conducted research on both [21, 37] (Table 2).

### SR

SR was reported in 13 studies [37, 39]. Most of the studies utilised the Kaplan–Meier estimated survival percentage. Six studies reported 5‐year SR [14, 20, 25, 33, 35, 40], one study reported 7‐year SR [13], 8 studies reported 10‐year SR [3, 5, 12, 20, 21, 25, 40, 42], 3 studies reported 15‐year SR [5, 20, 30]. One study reported a 16‐year SR [3], and a single study reported a 20‐year SR of 79,4% [12].

### 10‐year SR

Heterogeneity analysis was performed. The *I* ^2^‐statistics showed a high heterogeneity between studies. *I* ^2^ = 83%. A high heterogeneity suggests that the simple average is not representative of the overall trend; a pooled estimate was used.

**Figure 3:**
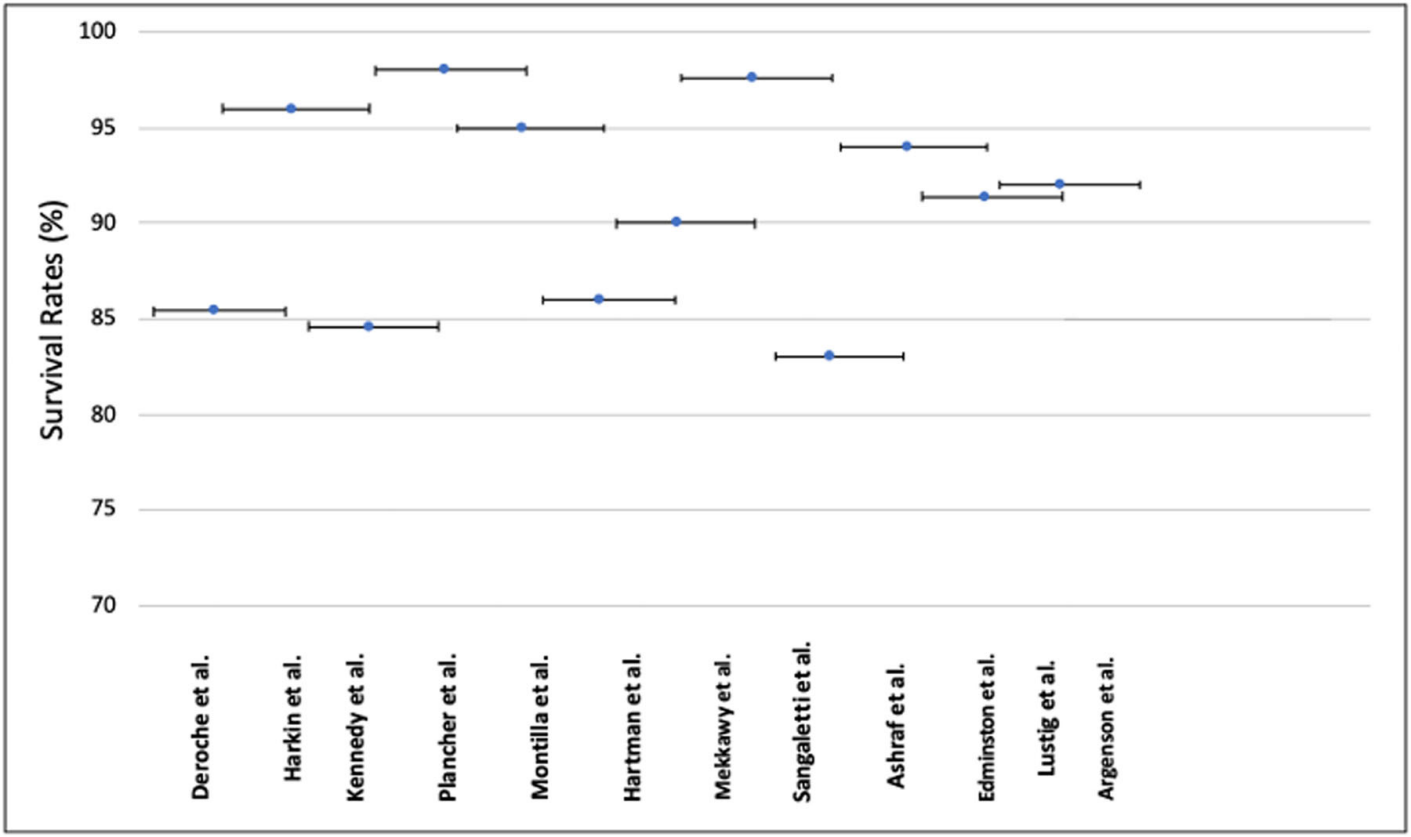
Forest plot of study 10‐year survival rates with 95% CI. CI, confidence interval.

The individual study weight, weighted SRs, pooled SRs and 95% CI were calculated (Figure 3). The pooled 10‐year SR was 93.7% (95% CI 92.5%−94.9%).

There was no correlation between the patient's age preoperatively and the SR (*p* = 0.06). The correlation coefficient (*r*‐value) is −0.18. A statistically significant negative correlation was found between patient BMI and SR (*p* = 0.03) (*r*‐value = −0.88).

There was a prevalence of female participants in all the studies of this review. The weighted mean male‐to‐female ratio was 0.453 (standard deviation [SD] = 0.20). A regression analysis was performed to investigate if the proportion of the male participants influenced the 10‐year SR. It ranged from 83% to 98%. The regression analysis revealed a slope of 0.23 and a y‐intercept of 0.83. This indicates that for every 10% increase in the proportion of males, the 10‐year survival rate increased by 2.3%. When *x* = 0 (indicating an entirely female population), the 10‐year SR was 83%. However, the *R* ^2^ value was 0.2, suggesting a weak relationship, as higher values closer to 1.0 denote a stronger association. Furthermore, the *p*‐value for the slope indicated no significant evidence of a strong association between the proportion of males and the SR (*p* > 0.05).

### Revision rates

From the collected articles, a total of 1562 LUKA procedures were made. A total of 133 revision surgeries were reported in total. The reported revision rate ranged from 0% to 17%. The expected rate was one revision for every 11th to 12th procedure. Two studies reported no revisions for 42 and 29 procedures, respectively [35, 39].

**Figure 4:**
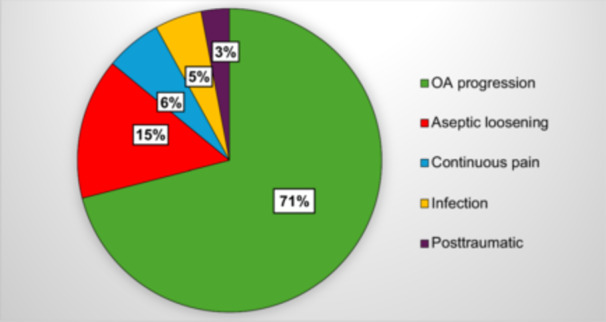
The most reported reasons for revision surgery after LUKA. LUKA, lateral unicompartmental knee arthroplasty; OA, osteoarthritis.

The main cause of revision surgery was the progression of OA. Other etiologies included continued pain, aseptic loosening, posttraumatic and infection (Figure 4).

**Figure 5:**
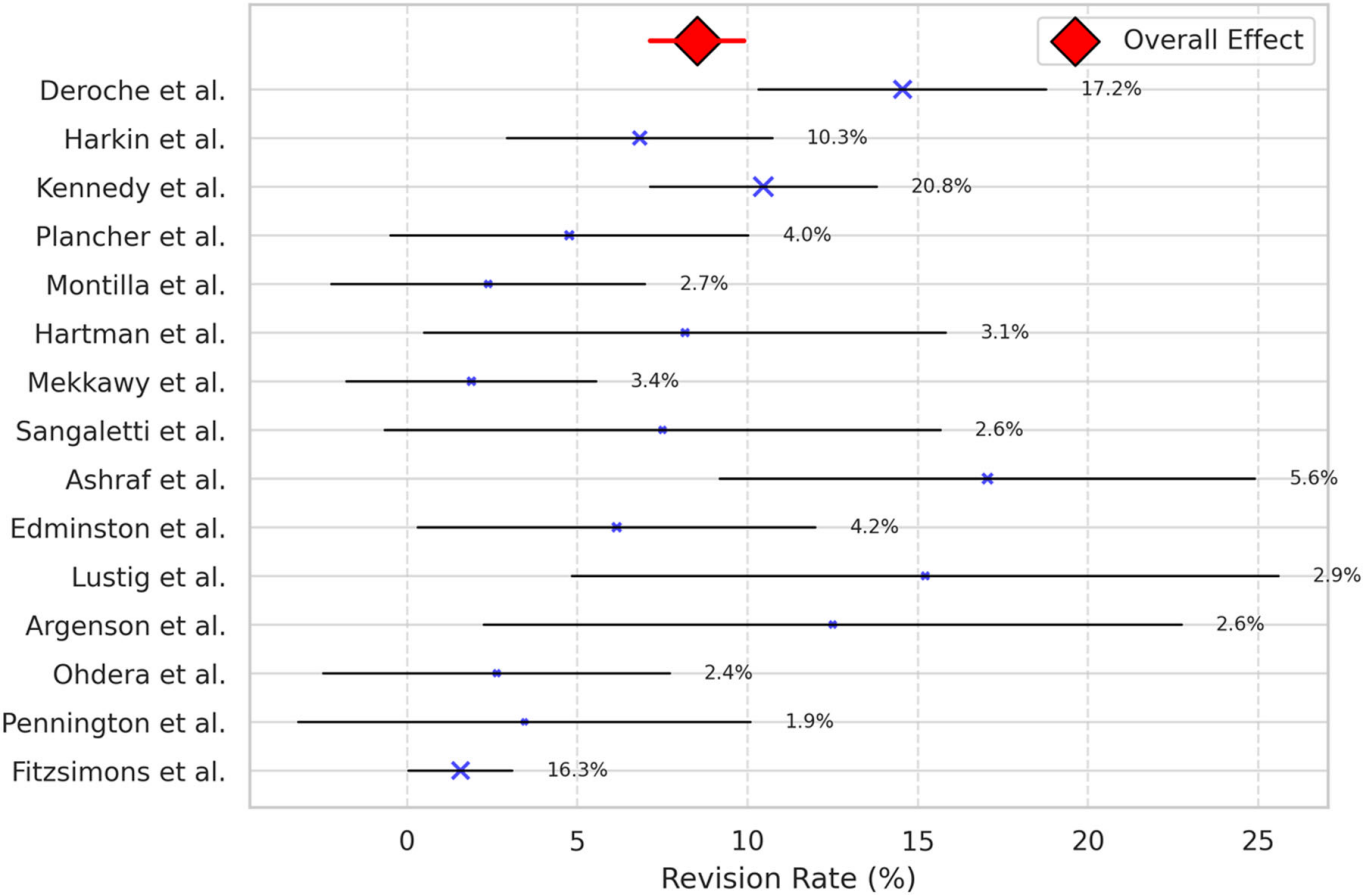
Forest plot, revision rates. The percentages on the right of each plot indicate the study weights.

**Figure 6:**
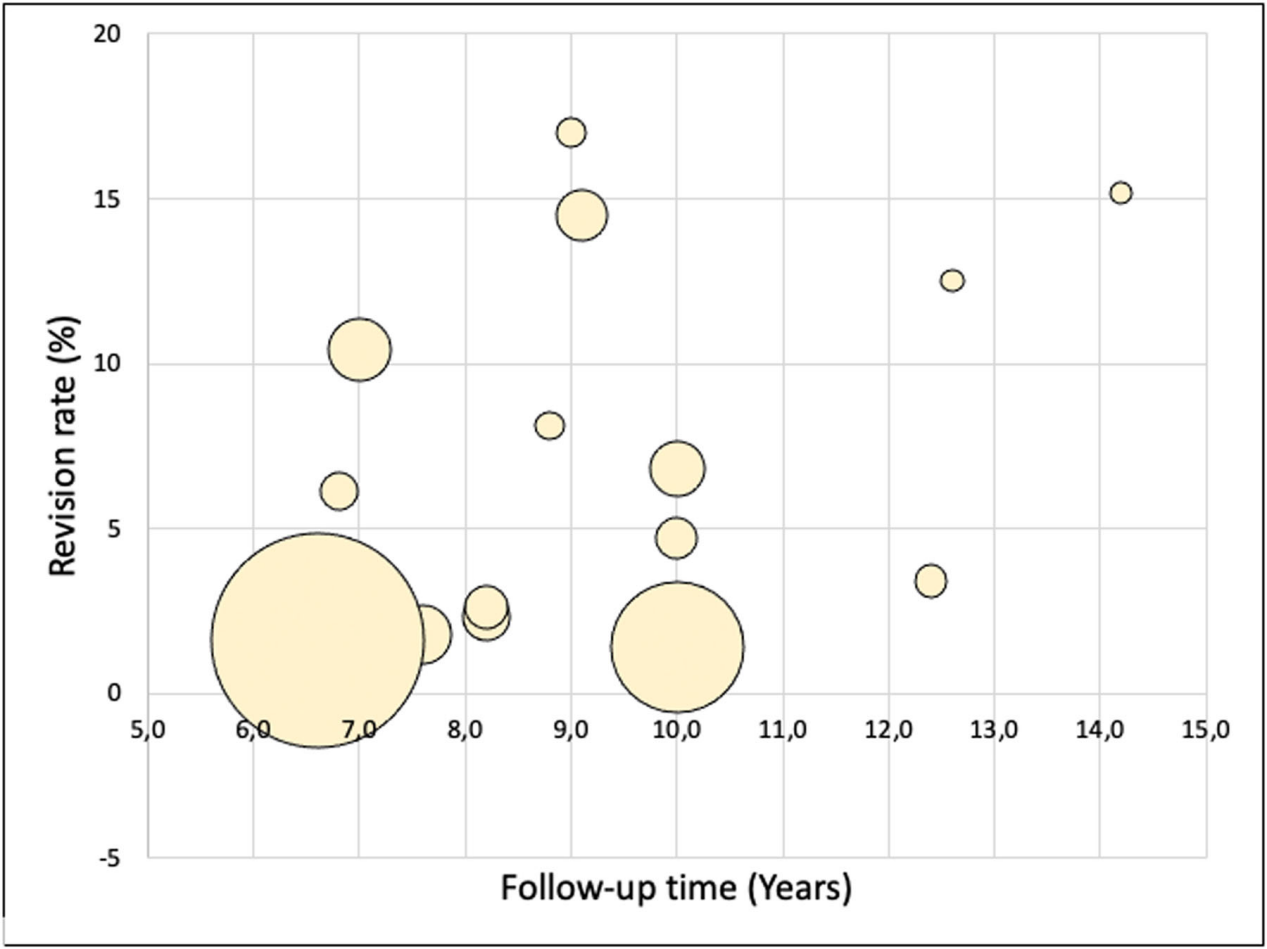
Bubble chart of the revision rates and study follow‐up. The size of the bubble represents the individual study weight.

The study heterogeneity for the revision rate was calculated. The *I* ^2^‐statistic was 49.4%, which indicated moderate heterogeneity, and a random‐effects model was used. The pooled estimate *R* was calculated. The overall revision rate was 4.6% (Figures 5, 6).

The studies utilised a total of 15 different tools for reporting outcomes, including both patient‐reported and clinician‐reported measures, alongside preoperative and postoperative ROM assessments. The scoring systems referenced included the Bristol knee score, forgotten joint score, hospital for special surgery (HSS) knee score, Japanese Orthopedic Association score, knee injury and osteoarthritis outcome score (KOOS), Knee Society Score (KSS), Lysholm Knee Score (LKS), 5‐point Likert scale, mean function score, Oxford Knee Score (OKS), single assessment numerical evaluation, Tegner activity score, UCLA Activity Score, visual analogue score and Western Ontario and McMaster Universities Osteoarthritis Index (WOMAC). Among these, the KSS [3, 13, 30, 35] and WOMAC [5, 14, 21, 42] scores were the most frequently used, appearing in four studies each. The OKS [5, 25, 42] and KOOS [20, 21, 40] were the second most commonly used measures, with three studies each reporting their results.

## DISCUSSION

This review found that LUKA consistently demonstrates high SRs and low revision rates in the long term, as evidenced by mid‐term results from other studies and reviews [6]. The procedure appears to be a reliable and well‐established option for managing lateral knee OA. The most important finding of this study, the pooled 10‐year SR for a lateral UKA procedure was 93.7%. Bonanzinga et al. reported a mean 5‐year SR of 95%, and a mean 5‐ to‐10‐year SR of 90% [7].

Van der List et al. compared the survivorship of medial and lateral UKA and found that the 5‐, 10‐ and 15‐year SRs for LUKA were 93%, 91% and 89%, respectively. There was no significant difference in SRs between medial and lateral UKA at 5 years (*p* = 0.71), 10 years (*p* = 0.88) and 15 years (*p* = 0.91). Additionally, the overall revision rate across 3296 procedures was 5.1% [29]. The overall revision rate from this study was comparable—4.6% (pooled estimate). The main reason for the revision was progression of OA. Other most common etiologies included continued pain and aseptic loosening.

Studies have demonstrated that knee surgeons who perform a higher volume of unicompartmental arthroplasty tend to achieve better patient satisfaction, as evidenced by patient‐reported outcome measures [34]. Additionally, subjective outcomes following UKA are influenced by psychological factors, highlighting the importance of optimal patient selection and appropriate indications for LUKA [28, 36].

The most reported specific indication for LUKA was a preoperative ROM of above 90 degrees, along with a correctable intraarticular valgus deformity, and a flexion contracture of less than 10 degrees. The main contraindications that the authors report were symptomatic PF OA, an ACL insufficiency, and a fixed valgus deformity. However, Kennedy et al. did not consider the state of the PF joint as a contraindication for LUKA [25]. This was reported in other publications as well [22]. An interesting finding was that only one study defined ‘asymptomatic PF OA’. It stated, ‘anterior knee pain on flexion or direct compression of PF joint’ [14].

Other indications that have been reported in the literature include posttraumatic arthritis due to a lateral tibial plateau fracture, and a postlateral meniscectomy lateral OA [32, 43]. Other contraindications that have been reported in the literature include an insufficient medial collateral complex, fixed deformities, poor bone stock and lateral bone loss [6]. Postdeformity correction may also be discussed as a potential contraindication. Postdeformity correction can alter knee alignment and joint loading, potentially affecting implant longevity. This, along with potential soft tissue compromise after correction, may elevate the risk of early implant failure [1].

Regarding patient‐specific indications, only Plancher et al. considered the patient weight as a factor in performing the procedure. A contraindication in their study was a BMI higher than 40 kg/m^2^ [40]. Baron et al. reported a BMI higher than 35 kg/m^2^ as a contraindication in an overview study [6]. In line with this, the meta‐analysis found a statistically significant negative correlation between patient BMI and SR (*p* = 0.03). On the other hand, Giordano et al. reported satisfying outcomes following LUKA in a BMI‐discrepant cohort. However, most of the patients in the study were overweight (BMI between 25 and 30), and the obese cohort had a mean BMI of 31, which could be argued borderline [17].

Additionally, it was discovered that females were significantly more likely to undergo a LUKA procedure for isolated lateral knee OA (*Z*‐score 6.87, *p* < 0.005). Possible explanations for female prevalence include biological factors, population demographics, culture or healthcare access factors. However, the regression analysis found that no strong evidence linked male proportion and SR (*p* > 0.05).

This review found that 79% of the studies reported that a fixed‐bearing implant type was used. This is in line with the concomitant trends and publications in orthopaedic literature [4, 48]. The most reported complication from a mobile‐bearing implant is revision due to bearing dislocation [26]. Hariri et al. discovered in a matched‐pairs analysis that the short‐to‐mid‐term implant SR of fixed‐bearing implants is statistically higher than the mobile‐bearing ones (78.7% at 3.4 years versus 98.3% at 2.7 years follow‐up) [19]. This trend is confirmed by this meta‐analysis's very high 10‐year pooled mean SR (>93%). On the other hand, Fornell et al. reported a 97.5% 5‐year SR with a mobile‐baring implant [15]. These discrepancies between studies may also be due to the surgical technique, as Zhang et al. reported surgical pearls on mobile‐bearing prostheses for over 500 operations [47]. This highlights the need for future randomised clinical trials comparing both designs.

Another tibia implant‐specific factor that has been reported in the literature is component external rotation. Excessive external rotation yields inferior results in patients undergoing a LUKA [16].

Regarding outcome reporting among studies, there were more than 10 different assessment measurements that were recorded throughout the articles. These included both patient‐reported and clinician‐reported outcome tools. Although most of them are famous and validated instruments for assessing knee function/patient satisfaction/quality of life, the variety among studies can be overwhelming. Therefore, a descriptive approach was taken in this article rather than quantifying the various results reported.

In line with modern advancements in arthroplasty surgery, the integration of robotic‐assisted and robotic‐navigated technologies in knee replacement warrants consideration. Robotic‐assisted LUKA has demonstrated high implant survivorship and long‐term patient satisfaction [41]. The use of robotic systems enables greater precision in the procedure, including improved bone cuts and a more standardised approach to LUKA, facilitated by the robot's preoperative planning capabilities. Surgeons input the relevant preoperative knee angles, allowing the robot to streamline the planning process. Additionally, comparable 5‐year survivorship rates have been reported between robotic‐assisted and conventional LUKA [31].

This study has several limitations. Because of an unclear or incomplete reporting of methods in some studies may result in their exclusion from the review, thus limiting the available data for the meta‐analysis and further conclusions. Additionally, some study heterogeneity existed, and pooled effects were estimated rather than the mean values. This may not represent the individual studies accurately. Not all results from studies were able to be combined, and therefore some descriptive statistics were utilised. Although study quality was assessed as high, the LoE across most selected studies was level IV, indicating a lower position on the evidence‐based ladder [45].

In conclusion, LUKA for isolated lateral knee OA provided a high long‐term SR with a low 10‐year revision rate. This was provided in a non‐ACL‐deficient knee with a preoperative flexion of above 90 degrees and a correctable valgus by using a fixed‐bearing implant. Females were more likely to undergo this procedure. A negative correlation was found between patient BMI and SR. There was a notable variability in the reported outcome measures among studies.

## AUTHOR CONTRIBUTIONS

All the authors contributed equally to the study.

## CONFLICT OF INTEREST STATEMENT

The authors declare no conflicts of interest.

## FUNDING INFORMATION

The authors have no funding to report.

## ETHICS STATEMENT

The Institutional Review Board (IRB) granted an exemption for this research. This study was registered in the PROSPERO register (ID Number: CRD42024618642).

## Data Availability

Data are available and may be obtained from the corresponding author upon reasonable request.
